# Improving Sleep Quality on the First Postoperative Night in the Intensive Care Unit: A Quality Improvement Initiative

**DOI:** 10.7759/cureus.94796

**Published:** 2025-10-17

**Authors:** Philippe Simard-Sauriol, Stéphanie Dupuis, Marie-Noëlle Côté, Myriam Boivin, Asaph Rolnitsky, Éric Camire

**Affiliations:** 1 Critical Care, Université Laval, Quebec, CAN; 2 Department of Anesthesiology and Critical Care Medicine, Centre Hospitalier Universitaire de Québec, Quebec, CAN; 3 Newborn and Developmental Paediatrics, Sunnybrook Health Sciences Centre, Toronto, CAN

**Keywords:** plan-do-study-act (pdsa), postoperative period, quality improvement (qi), sleep quality & quantity, surgical intensive care

## Abstract

Background

Sleep disruption in critically ill patients is common and associated with delirium, immune dysfunction, and reduced quality of life. The first postoperative night in the intensive care unit (ICU) is particularly challenging due to pain, anxiety, and frequent care. No intervention has yet reliably improved sleep for this population.

Methodology

We conducted a quality improvement project with six Plan-Do-Study-Act cycles over seven months. Postoperative adult patients admitted before 21:00 were eligible, with exclusions for instability or factors precluding reliable self-report. The primary outcome was patient-reported sleep quality (1-10 scale) the morning after ICU admission. Secondary outcomes included nurse-perceived sleep quality, sleep quantity, and patient satisfaction. Balancing measures included nurse workload and adverse events. Control charts were used to assess variation. Interventions included staff engagement, earplugs/eye masks, standardized door closure, pain management review, noise monitoring, simplified surveillance protocols, and melatonin.

Results

A total of 69 patients were recruited (23 pre-interventions, 46 during interventions). Patient-reported sleep quality increased from 5.0 to 5.4/10 after culture shift promotion. Nurse-perceived sleep quality had a favorable special cause variation after streamlined surveillance protocols. No increase in workload or adverse events occurred. The initial survey identified intermittent pneumatic compression devices as sleep disruptors, and the interim analysis showed that an epidural was associated with poorer sleep.

Conclusions

A multimodal, low-cost sleep bundle modestly improved sleep without increasing staff burden. Periodic data analysis supported real-time adaptation and may benefit similar initiatives. Future work should explore sustainability and impact on recovery.

## Introduction

Sleep disruption affects most critically ill patients and is associated with harm such as delirium, immune dysfunction, impaired glucose tolerance, and reduced long-term quality of life [1,2]. Contributing factors are classified as environmental (e.g., noise), physiological (e.g., pain, discomfort from devices), psychological (e.g., anxiety), care-related (e.g., analgesics), and preexisting sleep disorders [3]. No single intervention has reliably improved sleep in the intensive care unit (ICU), suggesting a need for individualized, multimodal approaches [4].

Sleep dysregulation is especially pronounced on the first postoperative night, a period marked by pain, intensive monitoring, and heightened anxiety [5,6]. In 2023, the Society of Anesthesia and Sleep Medicine emphasized the importance of postoperative sleep in recovery; yet, although some strategies have shown promise, no intervention has been conclusively validated in a diverse surgical ICU population during this high-risk period [3].

The ICU at Hôtel-Dieu de Québec (HDQ), a tertiary academic ICU admitting approximately 425 surgical patients annually, receives frequent patient complaints of poor sleep on the first postoperative night. A preliminary survey of 23 patients revealed an average self-reported sleep quality score of 5/10. Meetings with staff revealed multiple discrepancies between current practice and guidelines from the Society of Critical Care Medicine, highlighting opportunities for improvement [7].

Therefore, we undertook a quality improvement (QI) project to improve patient-reported sleep quality during the first postoperative night for ICU patients at HDQ. Our SMART (Specific, Measurable, Achievable, Relevant, Timely) aim was to increase the average self-reported sleep quality score from 5/10 to 7/10 over a seven-month period.

## Materials and methods

Setting

This project was conducted in a 15-bed tertiary academic ICU located in an urban center in Canada. The unit admits approximately 1,200 patients annually, with 30% undergoing major surgery. HDQ has expertise in complex oncologic surgical care, including general surgery (e.g., open lobar hepatectomy, Whipple), urology (e.g., open radical cystectomy and reconstruction), gynecology (e.g., hyperthermic intraperitoneal chemotherapy gynecological surgery), and ENT (e.g., regional flap reconstruction following cancer resection). All patients are cared for in private rooms with doors, and most rooms have large windows that allow natural daylight. The standard nurse-to-patient ratio is 1:2. Most patients receive publicly funded care with no out-of-pocket costs. Before the initiative, lights were routinely dimmed overnight, but door closure and pharmacological sleep aid prescriptions were inconsistent and left to caregivers’ discretion. The project was reviewed and deemed exempt from formal ethics approval in accordance with institutional guidelines for QI initiatives.

Improvement team

The QI team included two intensivists, an anesthesiologist, a surgeon, a critical care nurse, a patient partner, and a critical care fellow. The team met before each Plan-Do-Study-Act (PDSA) cycle to review data and adapt interventions. All decisions were guided by local feasibility, feedback from staff and patients, periodic statistical analysis, and key sleep disruptors identified at baseline.

Eligibility criteria

All postoperative adults (≥18 years) admitted to the ICU before 21:00 were eligible, ensuring they were exposed to the intervention throughout the full predefined sleep period (22:00-06:00). Exclusion criteria were medical instability (e.g., ≥0.1 µg/kg/minute noradrenaline), high-dose corticosteroids (>40 mg/day prednisone or equivalent), delirium at ICU admission, continuous parenteral sedation, mechanical ventilation, and major neurocognitive disorders. These exclusions were designed to keep confounding factors consistent and ensure patients could reliably self-report sleep quality.

Data collection tools

Two structured questionnaires were developed (see Appendices). Patient questionnaires were administered on the first postoperative morning, and nurse questionnaires were completed for every nurse who had responsibility for the patient between 22:00 and 06:00. Both questionnaires included quantitative ratings (e.g., sleep quality, quantity, perceived workload) and open-ended responses to capture qualitative insights.

Baseline assessment

**Figure 1:**
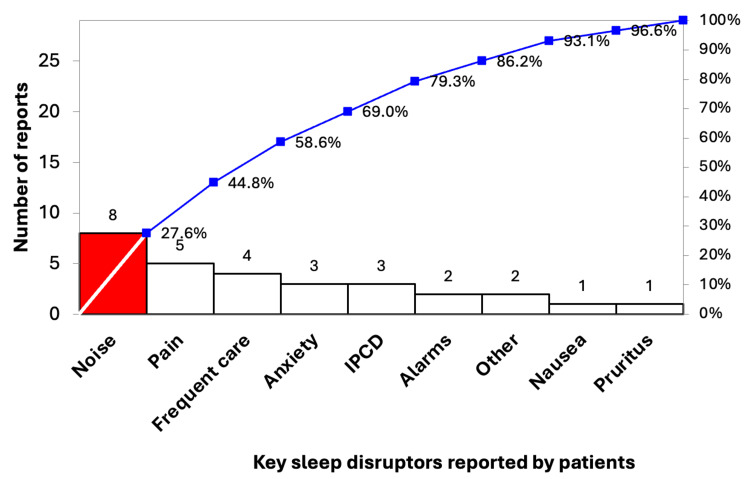
Pareto chart on key sleep disruptors reported by patients at baseline. Data are presented as the number of reports per key sleep disruptor and the cumulative percentage of reports. IPCD = intermittent pneumatic compression device

To understand baseline sleep quality and contributing factors, 23 patients were recruited before any interventions. Their responses informed a Pareto chart (Figure 1), identifying key sleep disruptors, namely, noise, pain, frequent care, anxiety, and intermittent pneumatic compression devices (IPCDs). Baseline data also enabled control chart development to assess process stability.

Interventions

Interventions were selected based on the Model for Improvement framework and adapted through iterative PDSA cycles conducted between June 17, 2024, and January 31, 2025 [8]. All new interventions were combined with existing ones to create a potentially more efficient care bundle. Details and a timeline are provided in Table 1.

**Table 1:** Timeline and description of PDSA cycles. PDSA = Plan-Do-Study-Act

PDSA cycle	Date	First patient to receive the intervention	Interventions
1	June 17, 2024	Patient #24	Raise awareness among caregivers and introduction of a performance board
2	July 01, 2024	Patient #31	Earplugs, sleep masks, reminder to close bedroom doors at night
3	July 04, 2024	Patient #32	Share pain data with anesthesiologists to raise awareness
4	August 20, 2024	Patient #39	Decibel meter
5	September 13, 2024	Patient #50	Simplified clinical monitoring protocol
6	January 30, 2025	Patient #68	Melatonin 3 mg before bedtime

In PDSA 1 (June 17, 2024), we displayed the Pareto chart and installed a performance board to increase staff awareness and promote a culture shift emphasizing sleep quality [3].

In PDSA 2 (July 1, 2024), we assured systematic provision of earplugs and eye masks, and standardized overnight door closure to reduce noise and light exposure [9,10].

In PDSA 3 (July 4, 2024), we shared data on pain complaints with our anesthesiology colleagues to encourage appropriate postoperative pain control. In our ICU, anesthesiologists prescribe initial pain medications for most patients, which are adjusted by ICU physicians as needed [11].

In PDSA 4 (August 20, 2024), informal interviews revealed excessive noise from the nursing station, especially during patient transfers. A decibel meter was installed to raise awareness [12].

In PDSA 5 (September 13, 2024), periodic statistical analysis revealed an association between epidurals and poorer patient-reported sleep quality. In collaboration with anesthesiology, surgery, and ICU leadership, we implemented a simplified monitoring protocol for stable patients, reducing unnecessary interventions [13] (see Appendices). The new protocol was not limited to epidural: it also reduced surveillance of drainage catheters, vital signs, glucose levels, and protocolized arterial line removal [14].

In PDSA 6 (January 30, 2025), we introduced low-dose (3 mg) melatonin before sleep, based on emerging evidence and updated Society of Critical Care Medicine guidelines [15].

Measures

Our primary outcome was patient-reported sleep quality (1-10 scale) on the morning after ICU admission. Our secondary outcomes were patient-reported sleep quantity, nurse-perceived sleep quality, and patient satisfaction (all three on a 1-10 scale). Nurse workload (1-10 scale) and descriptive report of interventions-related adverse events were included as balancing measures. Process insights were also captured through open-ended responses from patients and nurses.

To better understand contributing factors and support tailored interventions, we collected data on demographic characteristics (age and gender), potential modifiable risk factors (admission after 19:30, epidural, transfer of nursing care during the sleep period), and surgical specialty.

Statistical analysis

Primary and secondary outcomes were displayed using X-bar statistical process control (SPC) charts, created with QI Macros 2024 (KnowWare International, Colorado, USA) [16]. Evidence of improvement was assessed using the Institute for Healthcare Improvement SPC rules [17].

Preintervention and intervention phases were compared using a two-sided Student’s t-test or Wilcoxon-Mann-Whitney test for quantitative variables, and the exact Pearson chi-square test for categorical variables. The same tests, along with one-way analysis of variance F-test and Spearman correlations, were used to explore associations between outcome measures, potential modifiable risk factors, and specialty.

A maximum of one data point was missing per dataset. Given the small amount and the assumption that it was missing at random, we used mean imputation to replace these values. There were no missing data for the primary outcome.

All correlation and association analyses were conducted using SAS version 9.4 (SAS Institute Inc., Cary, NC, USA). A p-value <0.05 was considered statistically significant.

## Results

Participants

We recruited a convenient sampling of 69 patients between March 26, 2024, and January 31, 2025, with 23 in the preintervention phase and 46 in the intervention phase. Population characteristics and comparison of the population from both phases are presented in Table 2. There were no statistically significant differences between the populations of the two phases. Most patients were men (54%), and the median age was 69 years (interquartile range (IQR) = 59.0-73.0). Most patients had an epidural (78%), were transferred to another nurse during their sleep period (61%), and were operated on by general surgery (70%).

**Table 2:** Population characteristics, potential modifiable risk factors, specialty, and measures, overall and by recruitment phase. P-values are based on exact Pearson chi-square tests, except for age, which was assessed using the Wilcoxon-Mann-Whitney test. P-values <0.05 are considered statistically significant. P-values are not displayed for outcomes as change is meant to be determined by statistical process control chart interpretation rules. Data are presented as median (IQR), n (%) or mean (SD), as specified in the table. IQR = interquartile range; SD = standard deviation; ENT = ear, nose, and throat

	Overall (n = 69)	Preintervention (n = 23)	Intervention (n = 46)	P-value	Statistic
Demographic characteristics					
Age, median (IQR)	69.0 (59.0–73.0)	70.0 (63.0–75.0)	68 (57.0–73.0)	0.49	Z = 0.688
Women	32 (46%)	12 (52%)	20 (44%)	0.61	χ² = 0.466
Potential modifiable risk factors					
Admission after 19:30	19 (28%)	6 (26%)	13 (28%)	1	χ² =0.036
Epidural	54 (78%)	17 (74%)	37 (80%)	0.76	χ² =0.383
Transfer of care	42 (61%)	12 (52%)	30 (65%)	0.43	χ² =1.095
Specialty					
General surgery	48 (70%)	16 (70%)	32 (70%)	0.88	χ² =0.643
Urology	14 (20%)	4 (17%)	10 (22%)		
ENT	4 (6%)	2 (9%)	2 (4%)		
Gynecology	3 (4%)	1 (4%)	2 (4%)		
Outcomes					
Patient-reported sleep quality, mean (SD)	5.43 (2.72)	5.00 (2.30)	5.64 (2.91)	-	-
Patient-reported sleep quantity, mean (SD)	5.47 (2.32)	4.93 (2.07)	5.74 (2.41)		
Patient satisfaction, mean (SD)	9.53 (1.23)	9.41 (1.87)	9.59 (0.75)		
Nurse-perceived sleep quality, mean (SD)	6.59 (1.79)	6.28 (1.68)	6.75 (1.84)		
Nurse workload, mean (SD)	3.23 (1.67)	3.13 (1.48)	3.28 (1.77)		

A total of 111 corresponding nurse questionnaires were collected. Each patient had at least one associated nurse response, with some patients receiving care from two nurses across a single night due to handovers. In such cases, the nurses’ dependent outcomes were combined and prorated based on the number of hours each nurse was responsible for the patient during the sleep period.

Outcome measures

Primary Outcome

**Figure 2:**
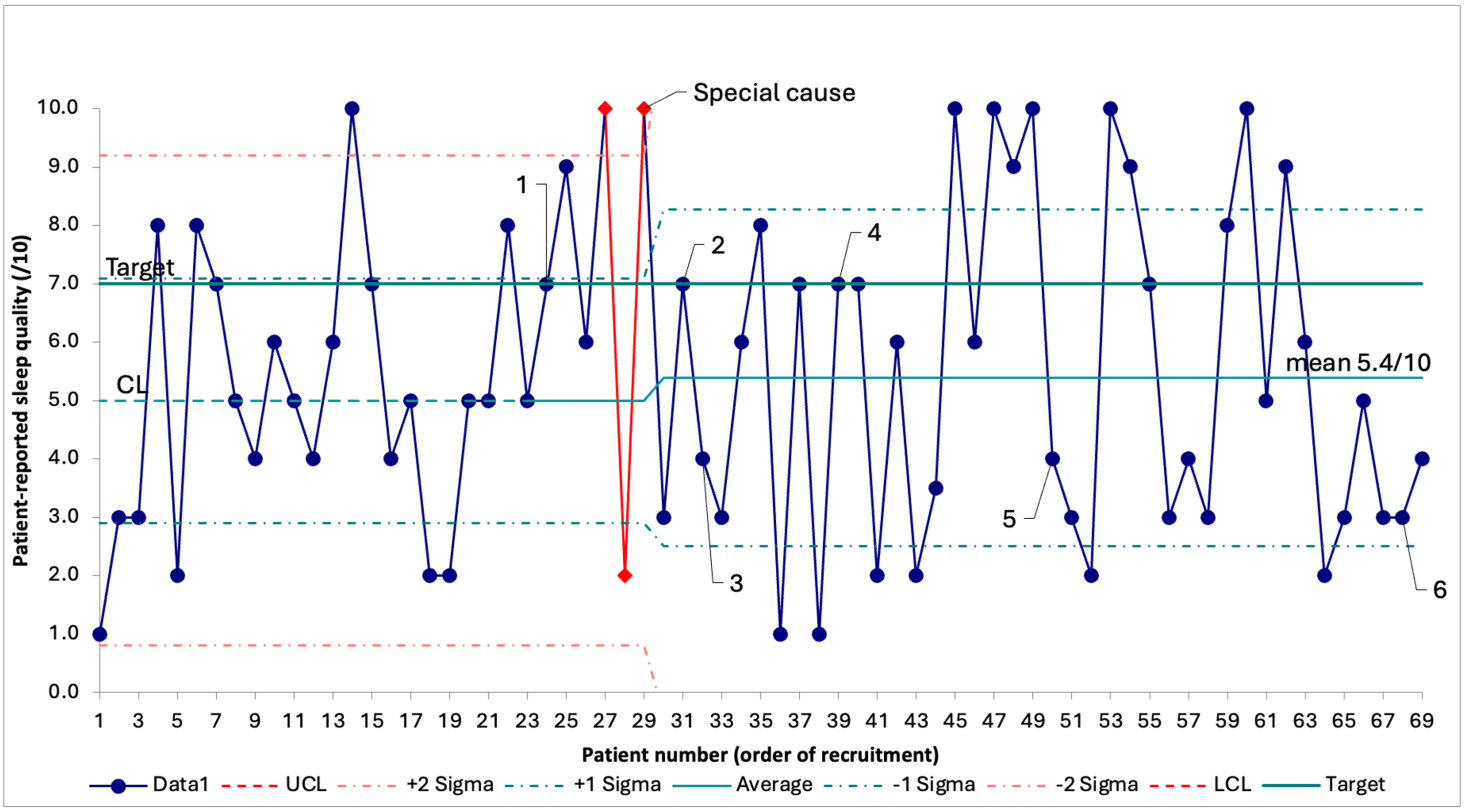
SPC chart on patient-reported sleep quality through recruitment. Data are presented as score results in order of recruitment. Number labels 1 through 6 represent the timing of the corresponding PDSA cycle. UCL and LCL are not visible because they fall outside the range of possible answers to the patient-reported sleep quality score. SPC = statistical process control; PDSA = Plan-Do-Study-Act; UCL = upper control limit; LCL = lower control limit

The mean patient-reported sleep quality increased from 5/10 to 5.4/10 after the implementation of the first PDSA cycle: special cause variation was detected through two out of three consecutive points falling inside the outer one-third of the chart (Figure 2). Updated mean and control limits were plotted in the SPC charts after a special cause was detected. After this process change, no further special causes were detected.

Secondary Outcomes

**Figure 3:**
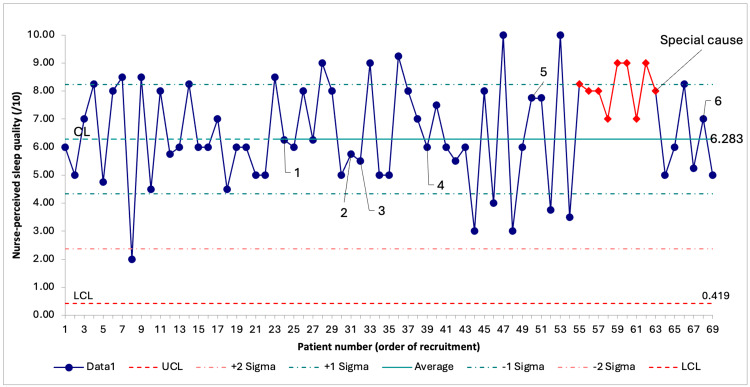
SPC chart on nurse-perceived sleep quality through recruitment. Data are presented as score results in order of recruitment. Number labels 1 through 6 represent the timing of the corresponding PDSA cycle. UCL is not visible because it falls outside the range of possible answers to nurse-perceived sleep quality score. As fewer than 12 data points were available after the special cause, a new process change could not be displayed. SPC = statistical process control; PDSA = Plan-Do-Study-Act; UCL = upper control limit; LCL = lower control limit

The mean nurse-perceived sleep quality was 6.59/10 (SD = 1.79), and a favorable special cause variation was detected after the fifth PDSA cycle: there was a shift of nine consecutive data points above the central line (Figure 3). As recruitment ended before we could gather 12 data points after the special cause variation, no conclusions can be drawn on the new process.

**Figure 4:**
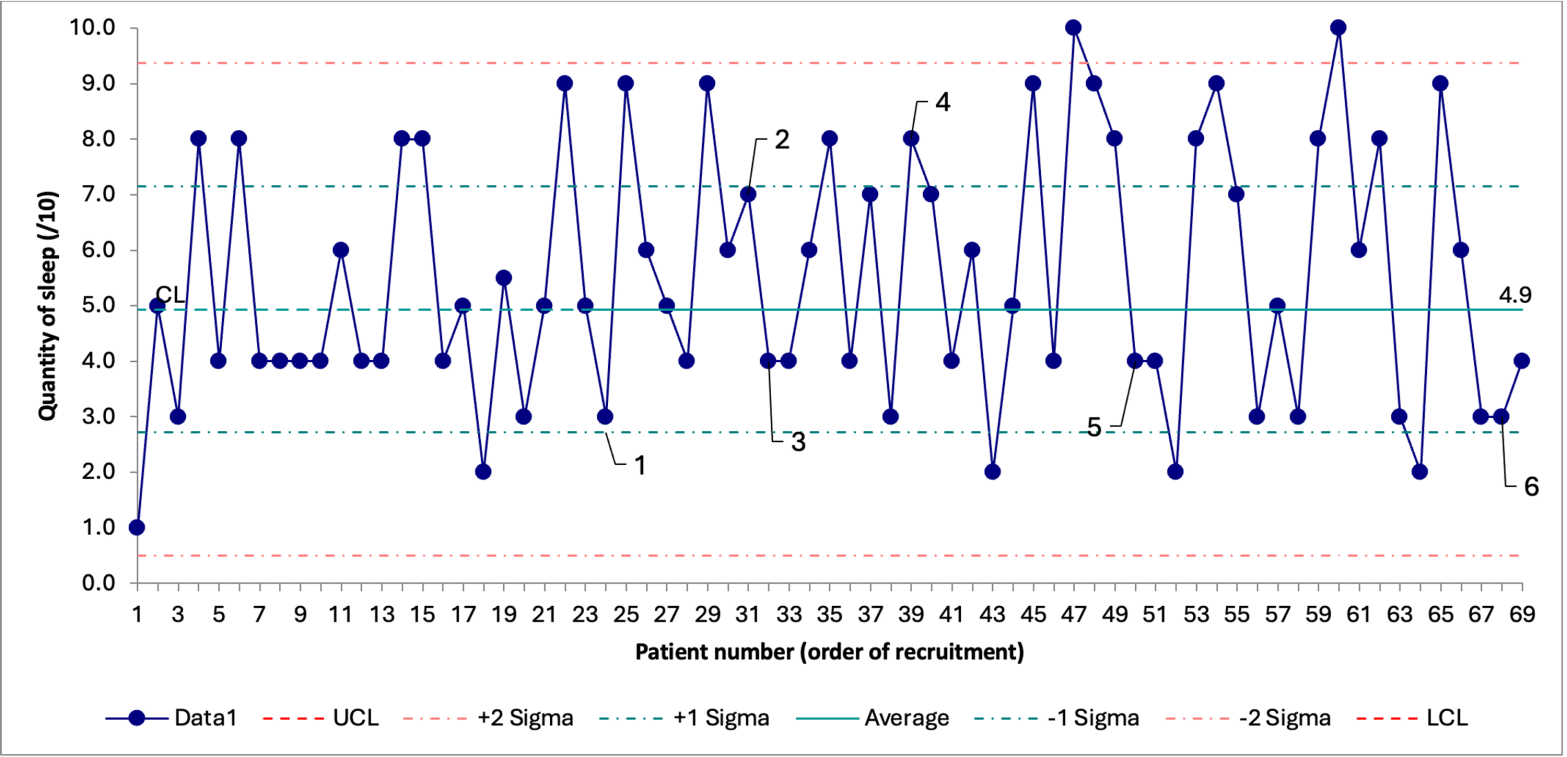
SPC chart on patient-reported sleep quantity through recruitment. Data are presented as score results in order of recruitment. Number labels 1 through 6 represent the timing of the corresponding PDSA cycle. UCL and LCL are not visible because they fall outside the range of possible answers to the patient-reported sleep quantity score. SPC = statistical process control; PDSA = Plan-Do-Study-Act; UCL = upper control limit; LCL = lower control limit

Patient-reported sleep quantity remained a stable process throughout the recruitment with a mean of 5.47/10 (2.32) (Figure 4). Early into the recruitment, seeing as the patient satisfaction score was nearly always 9 or 10/10, it became clear that this measure was a data for judgment rather than for improvement, and the QI team decided to discard it [16].

Balancing and Process Outcomes

**Figure 5:**
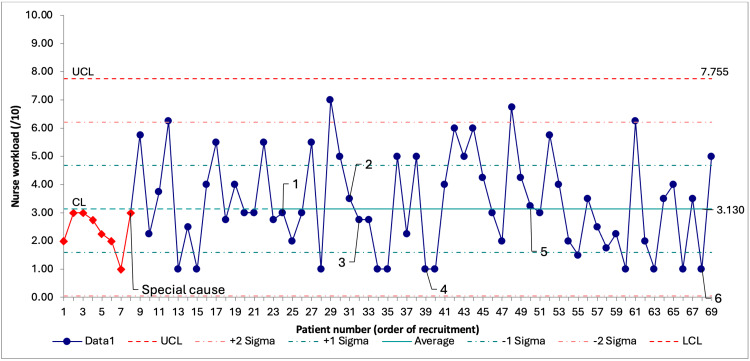
SPC chart on nurse workload through recruitment. Data are presented as score results in order of recruitment. Number labels 1 through 6 represent the timing of the corresponding PDSA cycle. LCL is not visible because it falls outside the range of possible answers to the nurse workload score. SPC = statistical process control; PDSA = Plan-Do-Study-Act; UCL = upper control limit; LCL = lower control limit

Nurses’ workload had a special cause variation involving the first eight data points: there was a shift of eight consecutive data points below the central line (Figure 5). It is unclear what contributed to this early variation. The process remained stable afterward, suggesting that interventions were well integrated into existing workflows.

No adverse events related to our interventions were reported. Process change did not require additional staffing or funding.

Demographic Characteristics, Potential Modifiable Risk Factors, Specialty, and Measures

Table 3 shows statistically significant associations. Better patient-reported sleep quality was associated with better patient-reported sleep quantity, urology, and absence of an epidural. Better patient-reported sleep quantity was positively associated with urology and negatively associated with ENT. Increased nurse workload was associated with transfer of care during the sleep period and the worst nurse-perceived sleep quality. No relevant associations were detected for age, gender, or admission after 19:30. Patient and nurse perception of sleep quality did not have a statistically significant association. These associations were identified during periodic statistical analyses to help the improvement team target effective new interventions and hold exploratory value.

**Table 3:** Statistically significant associations between measures, potential modifiable risk factors, and specialty. Only statistically significant associations (p < 0.05) are reported. P-values are based on Spearman correlation, Student’s t-test, or Wilcoxon-Mann-Whitney test, as specified in the table. Data are presented as correlation, mean ± SD or median (IQR), as indicated. SD = standard deviation; IQR = interquartile range; ENT = ear, nose, and throat

Variable 1	Variable 2	Test used	Result	P-value	Statistic
Patient-reported sleep quality (correlation)	Patient-reported sleep quantity	Spearman correlation	rₛ = 0.76	<0.001	
Nurse-perceived sleep quality (correlation)	Nurse workload	Spearman correlation	rₛ = -0.24	0.045	
Patient-reported sleep quality (mean ± SD)	Epidural (yes vs. no)	Student’s t-test	No: 6.9 ± 2.6 vs. yes: 5.0 ± 2.6	0.020	t = 2.392
Patient-reported sleep quality (mean ± SD)	Specialty (urology vs. others)	Student’s t-test	Urology: 6.9 ± 2.9 vs. others: 5.0 ± 2.5	0.020	t = -2.388
Patient-reported sleep quantity (mean ± SD)	Specialty (urology vs. others)	Student’s t-test	Urology: 6.7 ± 2.5 vs. others: 5.1 ± 2.2	0.020	t = -2.321
Patient-reported sleep quantity (median (IQR))	Specialty (ENT vs. others)	Wilcoxon-Mann-Whitney	ENT: 3.0 (2.5–3.5) vs. others: 5.0 (4.0–8.0)	0.020	Z = -2.417
Nurse workload (mean ± SD)	Transfer of care (yes vs. no)	Student’s t-test	No: 2.6 ± 1.4 vs. yes: 3.6 ± 1.7	0.010	t = -2.641

## Discussion

While we did not reach our target of increasing patient-reported sleep quality from 5 to 7 out of 10, this QI initiative still demonstrated improvement both with patient and nurse perceived sleep quality on the first postoperative night in the ICU. This was achieved through six low-cost, pragmatic interventions implemented over seven months, without increasing nurse workload or adverse events.

To our knowledge, this is the first initiative to target postoperative sleep on the first night in ICU across a broad surgical population using a multicomponent, context-specific bundle. While many studies have assessed sleep interventions in ICU settings, few have focused on this critical early postoperative period, despite the known vulnerabilities of these patients and the high prevalence of sleep disruption [18]. Our work adds to the emerging consensus that a multifaceted, behaviorally informed approach is required to improve sleep in critical care.

Key lessons

Several findings merit emphasis. First, we found that a visible culture shift, facilitated by publishing patient-reported barriers and creating a performance board, was associated with early improvement in patient-reported sleep quality, even before any material changes were implemented. This supports the hypothesis that organizational culture, staff awareness, and shared accountability can be powerful levers in quality improvement, echoing findings from other domains such as infection prevention and hand hygiene [19].

Second, we identified an unexpected but probable contributing factor: the frequent nighttime assessments required for patients with epidural analgesia. Despite the common belief that epidurals promote sleep through better pain control, our periodic statistical analysis showed an association between their use and lower sleep quality that persisted despite our efforts [20]. This may reflect confounding by indication, but the required intensive monitoring protocols during the first 12 hours may also contribute [21]. While our simplified surveillance protocol (PDSA 5) tried to mitigate this, further innovation is needed in this area, given how widespread epidural use is postoperatively.

Third, we found IPCDs to be among the key sleep disruptors. While the sleep-related impact of many monitoring and therapeutic devices has been studied, IPCDs have received little attention in this context [22]. Their benefit in preventing deep vein thrombosis is well established, but this evidence gap should prompt clinicians to reassess their continued use and remove them promptly when no longer clinically necessary [23].

Fourth, we found no statistically significant association between patient and nurse perceptions of sleep quality, contrary to findings in the general ICU population [24]. While this discrepancy may partly reflect differences in measurement tools, it may also highlight the unique challenges of assessing sleep in the immediate postoperative period, where pain and anxiety can obscure reliable evaluation [25]. While this uncertainty remains, caregivers should be cautious about assuming adequate sleep, especially when patient self-report is unavailable.

Lastly, the association between surgical specialty and sleep (e.g., with urology outperforming ENT) was likely confounded by care intensity. For example, ENT patients frequently required vascular flap monitoring, which is time-sensitive and impossible to cluster. Continuous Doppler monitoring was used; however, similar to many monitoring tools in our ICU, it lacked a remote alarm system and required frequent bedside assessments [26].

Strengths and limitations

Periodic statistical analysis allowed the QI team to better identify contributing factors and prioritize high-yield interventions. While the observed associations may have been attenuated by the improvement efforts themselves, they still offer meaningful insights to guide future QI initiatives.

Our use of open questions gave the QI team a unique perspective from both patients and nurses. We believe that this approach not only enhanced patient-centeredness but also helped build staff engagement and support, contributing to a broader culture shift [27].

While many QI initiatives target underdeveloped areas of care, we focused on a domain that, despite receiving considerable attention, continues to underperform in many otherwise high-performing ICUs [28]. We believed that tackling this complex issue was necessary to deepen our understanding and lay the groundwork for future improvement efforts. We are concerned by the scarcity of published QI initiatives reporting limited improvement, as these projects could offer critical insights into the most difficult problems in healthcare and be most relevant for well-developed care settings [29].

Several limitations should be acknowledged. First, our sample size was modest and drawn from a single center, limiting generalizability. Second, we used a convenient sampling subject to selection bias. Third, while more comprehensive tools such as the Richards-Campbell Sleep Questionnaire (RCSQ) exist, we relied on a brief, unvalidated 1-10 self-reported score. This decision was guided by ethical and practical considerations: both the head nurse and an ethics consultant emphasized the importance of minimizing patient burden and avoiding disruptions to rest and recovery on postoperative day one. Because our questionnaire also included other sleep-related items, it was not feasible to incorporate the full RCSQ without lengthening the survey and potentially disturbing recuperating patients [30]. Nevertheless, the consistency of associations and sensitivity to change suggest our measures were meaningful. Fourth, the improvement we observed was modest (from 5.0/10 to 5.4/10). While statistically significant, it remains uncertain whether this change is clinically meaningful or whether it would persist if a more robust, bias-limiting methodology, such as a randomized controlled trial, were used. Fifth, although we collected qualitative process insights through open-ended responses from patients and nurses, we did not obtain quantitative process measures due to limited resources, which restricts our ability to conclude that the observed improvement resulted from consistent application of the interventions. Finally, the initiative concluded shortly after melatonin was added to the care bundle because of limited resources, and this timing was not planned. As such, we cannot assess the impact of this intervention or the long-term durability of the broader process change.

Further work is needed to evaluate sustainability, assess generalizability to other centers, and determine whether improvements in sleep translate into better long-term recovery. The associations between sleep and various patient characteristics, such as types of anesthesia or pain medication, remain underexplored.

## Conclusions

This initiative offers a practical, evidence-informed framework for improving patient- and nurse-perceived sleep in surgical patients on their first postoperative night in the ICU. Despite their high risk for sleep disruption, limited improvement was achieved using simple, low-cost interventions co-designed with frontline staff. Periodic statistical analysis helped the team identify epidural as a contributing factor and refine the bundle over time, illustrating its value in adaptive, data-informed quality improvement. While further work is needed to evaluate long-term impact, elements such as culture change and simplified monitoring protocols could be readily adapted in other surgical critical care settings.

## Appendices

**Table 4:** Patient sleep quality and quantity questionnaire. English translation. Original in French.

Question	Scale/Response
What is your name?	_______________________________________
On a scale from 1 to 10, how would you rate the quality of your sleep last night, with 10 being the most restful sleep of your life?	1 – 2 – 3 – 4 – 5 – 6 – 7 – 8 – 9 – 10
On a scale from 1 to 10, how would you rate the quantity of your sleep last night, with 10 being an ideal duration of sleep?	1 – 2 – 3 – 4 – 5 – 6 – 7 – 8 – 9 – 10
On a scale from 1 to 10, how would you rate your satisfaction with the care you received in the ICU, with 10 being perfectly satisfied?	1 – 2 – 3 – 4 – 5 – 6 – 7 – 8 – 9 – 10
What obstacles interfered with your sleep?	__________________________________________
Do you have any suggestions to help us improve our patients’ sleep?	__________________________________________

**Table 5:** Nurse questionnaire – sleep quality and quantity in the intensive care unit. English translation. Original in French.

Section/Question	Response options
Eligibility – Patient: Does your patient meet all of the following conditions? - Postoperative after elective surgery - Admitted to ICU after 21:00 - Medically stable - Will likely be able to complete the patient questionnaire and sign consent after the first ICU night (only first-night data eligible) - Does NOT have any of the following: • Delirium • Major neurocognitive disorder • Intubated • Continuous sedation required • Prednisone >40 mg/day (or Solu-cortef >200 mg or dexamethasone >6 mg)	Yes/No
Eligibility – Nurse: Do you meet all of the following conditions? - You cared for the patient between 22:00 and 06:00 (at least part of this period) - This was your first night with this patient (only first-night data eligible)	Yes/No
What is your name?	__________________________
What is the date?	__________________________
What were your shift start and end times?	__________________________
What is the patient’s medical record number for whom you are completing this questionnaire?	__________________________
On a scale from 1 to 10, how would you rate your patient’s sleep last night, with 10 being the best sleep you have ever observed?	1 – 2 – 3 – 4 – 5 – 6 – 7 – 8 – 9 – 10
On a scale from 1 to 10, how would you rate your workload last night, with 10 being the busiest night of your life?	1 – 2 – 3 – 4 – 5 – 6 – 7 – 8 – 9 – 10
Between 22:00 and 06:00, was there a call to a physician for an unplanned prescription of a sleeping pill or additional analgesia?	Yes/No
What obstacles interfered with your patient’s sleep?	__________________________________________ __________________________________________
Do you have suggestions to help us improve our patients’ sleep?	__________________________________________ __________________________________________
Do you believe the proposed interventions (earplugs, eye masks, closing doors, etc.) were helpful for your patient?	__________________________________________
Do you believe the proposed interventions caused harm to your patient? (If yes, please contact: philippe.simard-sauriol.1@ulaval.ca)	__________________________________________

**Table 6:** Simplified monitoring protocol. English translation. Original in French.

Criteria needed to be met continually in the last 4 hours to apply the simplified monitoring protocol	
Patient eligibility	- Participation in the sleep improvement project in the ICU after elective surgery hospitalized in HDQ ICU
Neurological status	- GCS 15, no major cognitive impairment, no delirium
Hemodynamics	- SBP ≥ 90 mmHg and MAP ≥ 65 mmHg. Norepinephrine ≤ 0.1 mcg/kg/min without increase over the last 2 hours
Heart rate	- Between 50 and 110/min
Cardiac rhythm	- No new arrhythmia
Ventilation	- No ventilation (except CPAP or BiPAP for sleep apnea)
Urine output	- ≥30 mL/h (except dialysis patients)
Lactate	- <2.5 mmol/L (if measured PO)
Drainage	- All drains <50 mL/h each
Simplified monitoring	
Vital signs	- Measure BP, HR, RR every 2 hours ×2, then every 4 hours. - For patients without an arterial line on norepinephrine, monitor BP every 30 min
Temperature	- Every 8 hours
Arterial line	- Removal of arterial line if positional OR if BP more than 75 in the last 4 hours without vasopressor AND it causes discomfort AND no further intervention is planned AND no difficulty is anticipated for blood tests
Urine output	- Every 2 hours ×2, then every 4 hours
Drainage	- Drain output every 2 hours ×2, then every 4 hours
Pain assessment	- Evaluate pain score every 2 hours ×6, then every 4 hours
Regional analgesia	- For patients with epidural, continue monitoring motor block per anesthesia protocol (every 4 hours) and measure sedation score every 4h
Mobilization	- Do not mobilize patients between 22:00 and 7:00 unless specifically requested by the patient
Insulin protocol	- For patients with protocol CQ5733 and for whom a blood glucose test is scheduled for 1:00, perform it between midnight and 2:00, at the same time as other care or while the patient is awake
Monitoring	- Maintain continuous pulse oximetry and cardiac monitoring
